# Losing Much More Than a Transplant: A Qualitative Study of Kidney Transplant Recipients’ Experiences of Graft Failure

**DOI:** 10.1016/j.ekir.2024.07.011

**Published:** 2024-07-17

**Authors:** Anita Marie Slominska, Elizabeth Anne Kinsella, Saly El-Wazze, Kathleen Gaudio, M. Khaled Shamseddin, Ann Bugeja, Marie-Chantal Fortin, Mireille Farkouh, Amanda Vinson, Julie Ho, Shaifali Sandal

**Affiliations:** 1 MEDIC, Research Institute of the McGill University Health Centre, Montreal, Quebec, Canada; 2 Institute of Health Sciences Education, Faculty of Medicine and Health Sciences, McGill University, Montreal, Quebec, Canada; 3 Division of Nephrology, Department of Medicine, Queen’s University, Kingston, Ontario, Canada; 4 Division of Nephrology, Department of Medicine, Kidney Research Centre, Ottawa Hospital Research Institute, University of Ottawa, Ottawa, Ontario, Canada; 5 Centre de recherche du Centre hospitalier de l’Université de Montréal, Montréal, Quebec, Canada; 6 Alberta Cancer Foundation, Edmonton, Alberta, Canada; 7 Division of Nephrology, Department of Medicine, Dalhousie University, Halifax, Nova Scotia, Canada; 8 Department of Internal Medicine, University of Manitoba, Winnipeg, Manitoba, Canada; 9 Divisions of Nephrology and Experimental Medicine, Department of Medicine, McGill University, Montreal, Quebec, Canada

**Keywords:** descriptive methodology graft failure, kidney transplantation, loss, psychosocial support, transitions of care

## Abstract

**Introduction:**

Kidney transplant recipients with graft failure are a growing cohort of patients who experience high morbidity and mortality. Limited evidence guides their care delivery and patient perspective to improve care processes is lacking. We conducted an in-depth exploration of how individuals experience graft failure, and the specific research question was: “What impact does the loss of an allograft have on their lives?”

**Methods:**

We adopted an interpretive descriptive methodological design. Semistructured in-depth narrative interviews were conducted with adult recipients who had a history of ≥1 graft failure. Data were collected until data saturation was achieved and analyzed using an inductive and thematic approach.

**Results:**

Our study included 23 participants from 6 provinces of Canada. The majority were on dialysis and not waitlisted for retransplantation (60.9%). Our thematic analysis identified that the lives of participants were impacted by a range of tangible and experiential losses that go beyond the loss of the transplant itself. The themes identified include loss of control, loss of coherence, loss of certainty, loss of hope, loss of quality of life, and loss of the transplant team. Although many perceived that graft failure was inevitable, the majority were unprepared. The confusion about eligibility for retransplantation appears to contribute to these experiences.

**Conclusion:**

Individuals with graft failure experience complex mental and emotional challenges which may contribute to poor outcomes. The number of patients with graft failure globally is increasing and our findings can help guide practices aimed at supporting and guiding them toward self-management and adaptive coping.

## Graphical abstract

**Figure:**
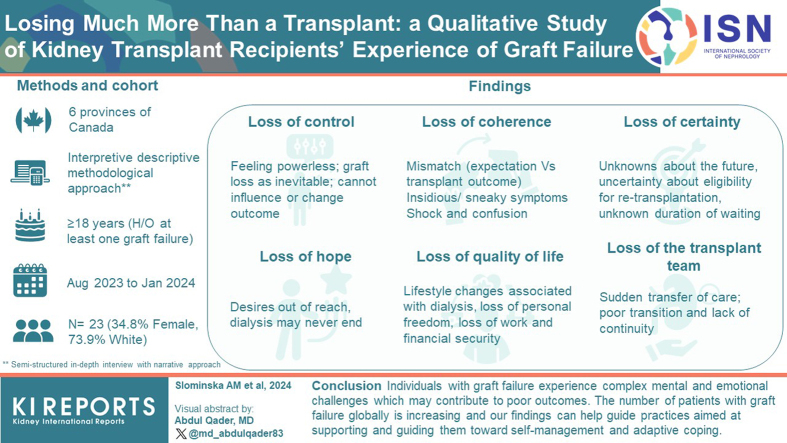

Although kidney transplantation is the ideal treatment for patients with kidney failure, many kidney transplant recipients experience graft failure.^1^, ^2^, ^3^ One in 5 patients is estimated to lose their graft within 5 years of transplantation and over half within 10 years.^2^^,^^3^ Their outcomes and quality of life are worse than transplant-naïve patients with kidney failure and mortality is disproportionately high in the first year after allograft loss.^4^, ^5^, ^6^, ^7^, ^8^, ^9^, ^10^, ^11^ Reasons postulated for these are multilayered and may be due to uncertainties regarding the care of these patients.^2^^,^^11^, ^12^, ^13^, ^14^, ^15^, ^16^, ^17^, ^18^, ^19^ Evidence to guide practices is conflicting and clinical care is described as fragmented.^5^^,^^6^^,^^20^, ^21^, ^22^, ^23^, ^24^, ^25^, ^26^ However, limited studies have explored the perspectives of those who experience graft failure to help guide care delivery and care transitions.^27^

The recent literature has highlighted the need for patient engagement as critical to preparing transplant recipients for different outcome trajectories after transplantation and pursuing patient-centered research on the experiences and psychological impact of the failing or failed graft.^26^^,^^28^ Among patients with native kidney failure, many studies have reported the profound psychosocial impact of their disease and of being on dialysis.^29^, ^30^, ^31^, ^32^, ^33^, ^34^, ^35^ These include decline in independence, self-esteem, and physical functioning, which contribute to depression and a deteriorating quality of life. Kidney transplantation allows for more freedom, autonomy and a return to regular activities compared to a lifestyle that is dictated by the constraints of dialysis.^36^ However, how patients experience the loss of this transplant, and other aspects of their life is not well-defined.

Psychosocial aspects of graft failure are likely profound with some of the literature describing it as a challenging and disruptive state that entails significant suffering and disenfranchised grief.^37^^,^^38^ Many patients report that their losses were not adequately recognized by their health care professionals.^38^ A better understanding of patient experiences can facilitate transitions of care between different health care teams, promote a positive experience, and lessen the psychosocial impact of graft failure. Thus, the aim of our study was to conduct an in-depth exploration of the experiences of kidney transplant recipients who experienced graft failure. In this paper, we explore the concept of loss, defined as “being unable to keep or maintain something or someone, either partially or completely.”^39^ Our specific research question was: “What impact does the loss of an allograft have on the lives of transplant recipients?”

## Methods

This study was approved by the research ethics board of the McGill University Health Centre in Montreal Canada. We followed the Consolidated Criteria for Reporting Qualitative Research guideline and checklist to ensure rigor in our reporting (Supplement 1).^40^

### Design

We adopted an interpretive descriptive methodologic approach. Interpretive description is a rigorous methodology suitable for researching clinical problems and collecting experiential knowledge with the goal of providing insight for the improvement of care.^41^ Herein, emphasis is placed on understanding the individual, their viewpoint, and their interpretation of the world around them, using iterative data collection and analysis.^42^ Our data collection focused on eliciting information about the needs of individuals experiencing graft failure and how they experience the loss of their transplant. Our study team included 2 patient partners with lived experience of transplant loss.

### Participant Eligibility Criteria

Adult kidney transplant recipients aged >18 years who spoke English or French and who had a history of at least 1 kidney transplant that lost function, necessitating another transplant or dialysis were eligible to participate. Participants who chose not to pursue either therapy or instead to pursue a conservative approach during graft loss, were also eligible. The transplant may have occurred in another country; however, the graft loss journey must have been experienced in Canada. This criterion was to ensure that lack of access to health care did not contribute to graft loss because Canada provides universal and publicly administered health care to the population. Participants were compensated with a $50 gift certificate in appreciation of their time and contribution.

### Recruitment

Purposive sampling was used to recruit participants who met the eligibility criteria of the study. A recruitment flyer was shared by participating transplant programs with eligible transplant recipients with graft failure. The flyer was also circulated by email by the Canadian Donation and Transplantation Research Program and posted on the social media pages of our team. The flyer explained the study, described eligibility, and provided contact details. Among those who contacted us, eligibility was determined based on participants’ self-reports. Snowball sampling techniques were also used.

### Data Collection

Semistructured in-depth interviews that used a narrative approach were conducted with eligible participants. These interviews were conducted between August 2023 to January 2024 by a female qualitative researcher with PhD training and over 10 years of experience (AMS). The participants had no previous relationship with the interviewer. Participants were aware of the interviewer’s credentials, and it was explained that the purpose of the study, and the interviewer’s interest, was to understand their experience. Interviews lasted between 32 and 112 minutes and were conducted remotely and privately over the telephone or via the Zoom platform. Participants were interviewed once. Open-ended questions were used to elicit participants’ narrative accounts, followed by questions to elaborate on issues of interest corresponding to the objectives of the study. The goal of the interviews was to understand how participants experienced graft loss, investigating the psychosocial aspects as well as the details of their health care experience. The interview guide was piloted with 6 participants and revisions were made with feedback from the study team, including the patient partners (Supplement 1). The interviewer took notes and wrote brief memos during and after the interviews to foster reflexivity, identify potential follow-up questions, and record key points. All interviews were recorded and transcribed verbatim. Transcripts were verified by 1 member of the study team (AMS), but they were not returned to participants for comments or corrections.

### Data Analysis

Several techniques were used to conduct a systematic and thorough inductive analysis. Each transcript was closely read by 2 study team members (AMS and SEW) who independently created a mind map for each individual interview. Mind maps were used to inductively identify central concepts and document them in a diagram.^43^^,^^44^ The researchers met regularly to compare mind maps, to discuss and harmonize key emergent ideas, and to produce a table summarizing themes and subthemes for each interview. Using constant comparison approach, we noted interpretive patterns in the summary tables and an overall thematic analysis emerged.^45^ Simultaneously, transcripts were line-coded by 2 researchers (AMS and SEW) using Quirkos (Edinburgh, UK), a qualitative analysis software. The data were organized into relevant categories to support interpretation and analysis and to ensure that the themes were grounded in and traceable to the raw data. A codebook was developed iteratively and collaboratively by the 2 coders using the software’s project sharing tools. Data saturation was achieved when additional data yielded no new insights, and the data were seen to support recurring themes.^46^ The identified themes were presented to the wider research team, including patient partners, for their feedback but member checking was not pursued.

## Results

### Sample

Of the 27 individuals who contacted us and met our eligibility criteria, 23 transplant recipients who had experienced graft failure participated. The other 4 either expressed noninterest or did not reply to requests to schedule an interview. The participants were from 6 provinces across Canada, 34.8% identified as female, and 73.9% identified as White recipients. Participants reported different outcome trajectories after graft loss as follows: 17.4% received another transplant; 17.4% were on the waitlist for another transplant while being on dialysis; 60.9% were on dialysis but not on the waitlist; whereas for 4.3%, their status was not available (Table 1). Thematic saturation was achieved with 23 participants: we had collected sufficient supporting data for our themes and no new themes were emerging.

**Table 1:** Sociodemographic characteristics of participants

Age (mean yr and range)	54.3 (31–75)
Women (%)	34.8
Race or ethnicity	
White	73.9
Haitian	8.7
Mixed	4.3
West Indian	4.3
Chinese	4.3
Not reported	4.3
Province of residence (%)	
Alberta	13.0
Manitoba	8.7
New Brunswick	4.3
Nova Scotia	13.0
Ontario	30.5
Quebec	30.5
Level of education (%)	
High School	21.7
Some college/college diploma	30.4
Some university/university degree	34.7
Not reported	13.2
Employment status (%)	
Full-time	26.1
Part-time	8.7
Disability Leave	26.1
Unemployed	8.7
Retired	26.1
Not reported	4.3
Language used during interview (%)	
English	82.6
French	17.4
Current status (%)	
Retransplanted	17.4
On dialysis and waitlisted	17.4
On dialysis and not waitlisted	60.9
Not available	4.3
Age at transplant (mean and range)	36.9 (10–71)
Age at transplant loss (mean and range)	44.5 (20–71)

**Figure 1:**
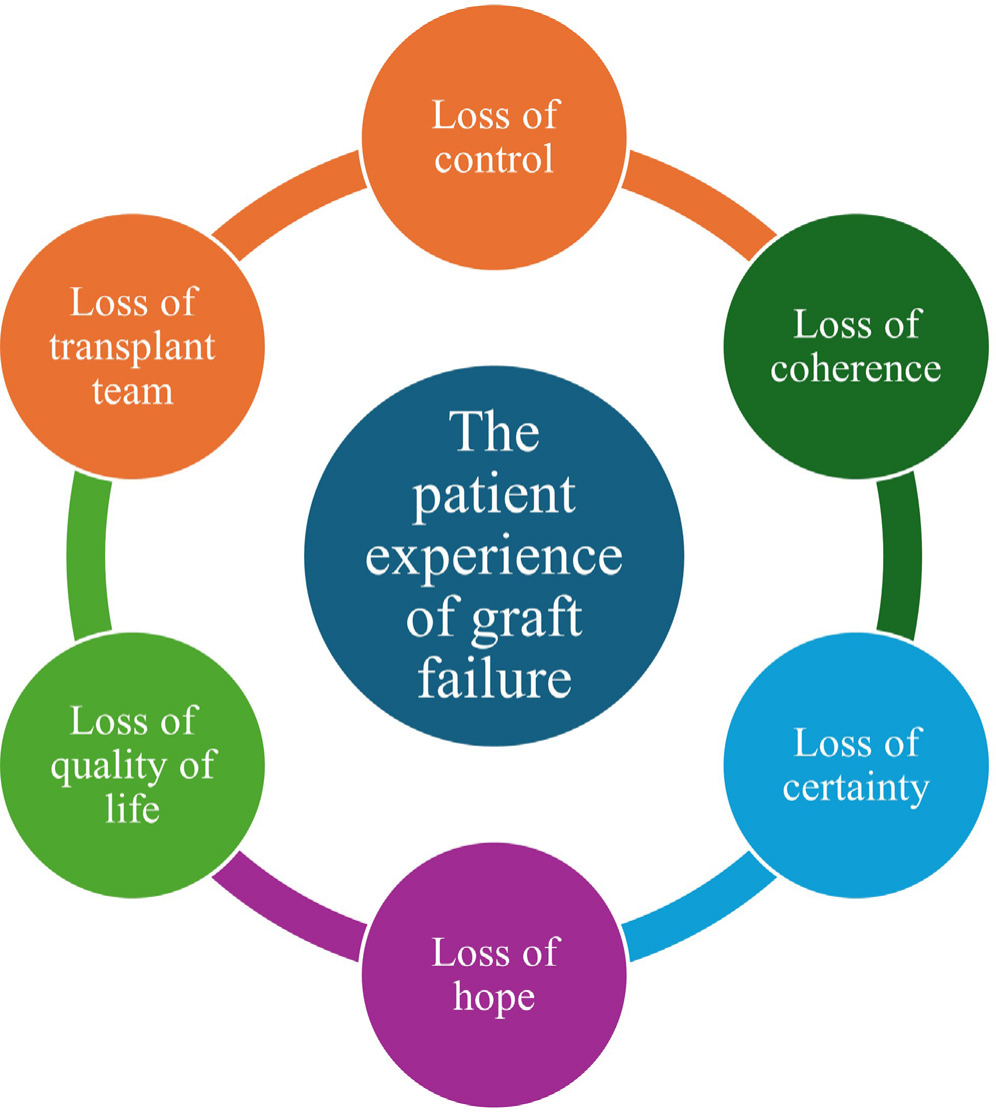
The range of tangible and experiential losses experienced by kidney transplant recipients with graft failure.

Our thematic analysis identified that the lives of individuals with graft failure are impacted by a range of tangible and experiential losses that go beyond the loss of the transplant itself. Although each individual person’s experience is shaped by their own perceptions and circumstances, we identified 6 types of losses that represent concrete and conceptual descriptions of recipients’ lived experience, including how individuals in the study contended with different types of loss, and the grief and emotions that accompany them (Figure 1). These losses are described in the next section and representative quotes from participants are presented in Supplementary Table S1.

### Loss of Control

Most participants described that the loss of the transplant was ultimately inevitable and expressed feelings that nothing in their power could have changed the outcome of graft failure. This included perceptions that the graft had simply run its course, or the attribution of loss to unforeseeable circumstances or misfortune (“luck of the draw”). Except for a few participants who viewed health provider’s mistakes or poor judgement as having caused or expedited graft failure, participants shared perceptions that the transplant team did everything they could and ultimately could not prevent graft loss from occurring. For many participants, knowing that “there’s no stopping your kidney failure*”* was a difficult psychological state to be in and that led to anxiety. Many also reported having a difficult time coming to terms with accepting that they had “no choice” but to return to dialysis. Loss of control was also present in the pursuit of retransplantation; many participants described having little influence over the process and were obligated to wait for the health team evaluation to determine whether they had been “accepted” as a candidate.

### Loss of Coherence

Graft loss entailed many elements of incoherence, and instances of dissonance, strangeness, and contradiction. This included an experience of incoherence that occurred with the sudden loss of a short-lived graft and entailed a stark mismatch between expectations of transplant success and duration, and outcomes that often led to shock and confusion. This occurred for those who went into transplant surgery thinking that graft failure was “rare” and unlikely to happen. One participant described the sudden loss of their transplant as “not a normal turn of events.” In some cases, participants felt that it was clinicians who amplified their expectations because they promoted the benefits of transplantation and downplayed “the negatives.”

In those with gradual graft loss, loss of coherence appeared in more subtle incongruities, namely in recipients reporting that they felt both prepared and not prepared for graft loss, or aware and not aware of graft failure. This resulted in some participants describing the loss of their transplant as not a “surprise” but also a “shock.” In some cases, physical symptoms were absent (made evident only in bloodwork); others described them as “insidious” or “sneaky.” Some explained that this resulted in not realizing how bad one was feeling because it happened incrementally (1 participant compared it to “boiling a frog”). Psychological factors such as the lack of acceptance and denial also contributed to loss of coherence: as 1 participant said, “you trick yourself into thinking that you are better than you are.”

### Loss of Certainty

Participants described significant uncertainty about the future. Contemplating going back on dialysis led to questions such as “how am I going to continue to do the things that I do?” Many reported difficulties imagining or planning for the future. One participant described the experience of losing their transplant as feeling “rudderless in an open sea.”

The possibility of relisting for retransplantation played a determining role in the effect of graft loss on their lives and not knowing of their chances led to profound uncertainty. Most were aware that eligibility depended on health status, and that poor health and age are barriers to retransplantation. The evaluation process took months, and this led to periods of doubt and concern about the next stages of illness and treatments. Participants who were aware that they were a “high-risk candidate” experienced more uncertainty than those who were told they were “a great candidate” for retransplantation. Some participants were uncertain about whether they wanted to pursue retransplantation and took time to reflect and question whether it would be worth it. Even when they were waitlisted for retransplantation, uncertainty still loomed given the unknown duration of waiting, particularly if they were immune sensitized; the awareness that any health setbacks could compromise their eligibility; and that a positive outcome was not guaranteed. Given these uncertainties, participants sometimes expressed the perception that everyone, including physicians, was “in the dark.”

### Loss of Hope

Many participants expressed a loss of hope and the feeling that their desires were out of reach. This included grieving for things they used to be able to do or longing for what they had but could not have anymore. Framed in this way, hope was interlinked with the themes of loss of control and loss of certainty. Graft loss could be understood as a “threat to hope” due to the way it erodes a sense of control and certainty about the future.

Most located hope in the possibility of a future successful retransplant (receiving a “pristine kidney”) even if they were aware that transplants are not a “permanent fixture” and they “don’t last forever.” Even a minimal amount of hope for another transplant seemed to make a difference with 1 participant describing it as a “little string to hang on to”. Loss of hope appeared most profound for participants who perceived that dialysis may never end and that they were approaching their “expiry date.” Some participants shared that they had considered stopping dialysis and giving up. To cope with the loss of hope, many participants underscored the benefits of positive thinking, taking a more pragmatic attitude of “carrying on” or accepting the “hand you are dealt with,” and reminding themselves that they have “the strength to deal with bad things.”

### Loss of Quality of Life

For most participants, the return to dialysis was inextricable from the graft loss experience. They expressed how it had a great impact on their day-to-day life and entailed lifestyle changes that negatively affected their quality of life. For some, it was the loss of work, livelihood, and financial security that had a severe consequence and precipitated stress due to hardships, such as food insecurity. Participants often framed the return to dialysis as losing a sense of “normalcy.” Transplantation was characterized as a “lifeline to the normal world” whereas the routine of dialysis was frequently described as outside the realm of “normal” life and as leading to a sense of only quasi-living (“not really living”). All the restrictions associated with dialysis deteriorated their quality of life by depriving them of a sense of freedom, as illustrated with phrases such as “tied to a machine” or a metaphorical “leash,” and even “jail.”

### Loss of Transplant Team

When care was transferred to a dialysis unit, the transfer of care was often described as sudden or abrupt. The lack of contact and communication with the transplant team after graft failure was sometimes depicted as having the psychological impact of feeling abandoned. “They wipe their hands off you,” 1 participant said; whereas another described it as being “kicked to the curb.” Others noted that the handoff and the absence of the transplant team were “cold,” such as the transplant team had moved on and “you are no longer their concern.” By contrast, some participants described feeling more supported when there was continuity of care and follow-up with the transplant team. Another difficulty of this transition was expressed as the contrast between the more personalized care they received as a “transplant patient” and the experience of being a “dialysis patient” where they felt more “like a number” and were seen by multiple doctors on rotation in the dialysis units. For most participants, the dialysis team was not involved in the process of seeking retransplantation and this fragmentation meant that participants did not have access to clear and coordinated communication with the transplant team.

## Discussion

In this interpretive-descriptive qualitative study, we gathered and analyzed experiential knowledge from individuals who experienced graft failure and report that they experience manifold losses in addition to losing their transplant that have psychosocial, functional, and physical implications in their lives. Loss of control and loss of quality of life emerged as major themes. Although many perceived that graft failure was inevitable, the majority were unprepared and reported loss of certainty and loss of hope. Loss of coherence based on the graft failure trajectory and loss of the transplant team were novel findings suggesting the need for better communication and support as patients transition to different outcome trajectories. To our knowledge, this is the first study that has probed deeply into the concept of loss experienced by recipients with graft failure.

Some dimensions of loss are similar to previous findings reported by patients with kidney failure, including loss of control and loss of quality of life.^31^^,^^47^, ^48^, ^49^, ^50^, ^51^, ^52^, ^53^, ^54^, ^55^, ^56^ The quality of life issues raised by our participants includes loss of personal freedom, functional ability, and capacity to fulfill social roles.^48^, ^49^, ^50^ Patients regard transplantation as a means of regaining control of their lives and being able to engage fully in preferred activities,^57^ and the loss of the transplant has been described as a loss of their previous life and imagined future.^38^ Our findings about these 2 losses corroborate the existing literature on patient experience of dialysis and transplantation.

However, our study broadened these findings and elucidated the constellation of other losses that those with graft failure experience that are unique to this population, such as the loss of the transplant team. Transplant recipients require specialized, integrated, and multidisciplinary care with a team approach to secure the benefit of transplantation and ensure optimal outcomes and adherence.^58^, ^59^, ^60^ Although there are differences in the structure and coordination of care between kidney transplant centers,^61^ the overall care of recipients is facilitated by the transplant coordinator who is often their point of contact.^62^, ^63^, ^64^, ^65^ We highlight the need for better support mechanisms as patients transition through different health care teams. Loss of coherence during the experience of a failing graft was also a unique finding and we found this to be linked to patient expectations about the outcome of their transplant. Previous studies have noted that recipients are often not well-informed about variable trajectories after transplantation, indicating a need for better preparation and counselling for outcomes such as graft failure.^47^^,^^55^^,^^66^, ^67^, ^68^, ^69^, ^70^

A key point raised by many participants was about retransplantation. Retransplantation is known to offer a survival advantage for eligible patients with graft failure, and a better quality of life.^71^, ^72^, ^73^ However, the confusion about eligibility contributed to the experiences described above, particularly the loss of certainty and the loss of control. Thus, a key implication of our findings is better communication and transparency about retransplantation criteria and candidacy.^55^^,^^66^

Our study is at risk of self-selection bias given our recruitment strategy and language restrictions. We are aware that voluntary participation may lead to a skewed representation of the broader population. Our study identified themes that had sufficient supporting data from our sample. However, we are cautious about the generalizability of our data across the spectrum of the patient population. For example, most participants in our study had postsecondary education and identified as White. We also had fewer women participants. Furthermore, we could not recruit a participant who decided to pursue supportive care. Collecting data through interviews is also at risk of response bias, meaning that participants may tailor their responses according to fear of judgment and social desirability. We mitigated this risk by employing an open and nonjudgmental interviewing style, including techniques of active listening and continual validation of the worthiness of each individual’s perspective, which are aimed at increasing participants’ comfort in sharing thoughts and experiences that are genuine and authentic. Finally, some of our findings may not be generalizable to other health systems with variable payment models.

Nevertheless, our findings have implications for the broader nephrology community because the absolute number of kidney transplantations being performed has increased significantly from 63,721 in 2004 to 102,149 in 2023.^74^ Simultaneously, the proportion of recipients experiencing graft failure is also increasing. Patient perspectives captured in our study underline the need for care practice improvement about graft failure. The first and most evident is the need for more robust and tailored psychosocial services for recipients. The loss themes that we identified are evidence that patients experience complex mental and emotional challenges with the loss of their graft, which may explain why many experience high morbidity and mortality after graft failure. These themes also suggest potential barriers and facilitators to adaptive coping that could help develop pertinent approaches to psychological support and counselling for transplant recipients. The findings from this study also provide indication of how to guide improvements to physician-patient discussions about graft failure. They may help physicians and health care providers in conducting conversations about experiences of loss with patients, to normalize patients’ experience of loss as a potential outcome of graft failure, and to sensitize physicians as to when appropriate referrals to other disciplinary specialists in grief and loss work such as social, palliative care, psychology, or spiritual care may be warranted.

Overall, the interpretation and analysis of our findings highlight the importance of loss as a theme across the spectrum of the transplant recipient experience and we pinpoint graft failure as a moment in the patient’s journey when it is particularly pronounced. By categorizing those losses as loss of control, loss of coherence, loss of certainty, loss of hope, loss of quality of life, and loss of the transplant team, we hope that our findings will contribute to knowledge about patient experience and lead to the development of better strategies to support and prepare patients for the outcome of graft failure and guide them toward self-management and adaptive coping.

## Disclosure

SS has received an education grant from Amgen Canada to improve the care of patients with graft failure. All the other authors declared no competing interests.

## Supplementary Material

Supplementary File (PDF) **Supplement 1.** Interview guide. Table S1. Themes and representative quotes. **COREQ Checklist.**

## Footnotes

Supplementary File (PDF)
**Supplement 1.** Interview guide. **Table S1.** Themes and representative quotes.
**COREQ Checklist.**
