# Supplementary material for: Losing Much More Than a Transplant: A Qualitative Study of Kidney Transplant Recipients’ Experiences of Graft Failure
Source: Kidney Int Rep. 2024 Jul 17;9(10):2937–45. doi: 10.1016/j.ekir.2024.07.011 (PMC11489391; doi:10.1016/j.ekir.2024.07.011)
Supplement: Supplementary File (PDF) — Supplement 1. Interview guide. Table S1. Themes and representative quotes. COREQ Checklist. [file mmc1.pdf]

COREQ (COnsolidated criteria for REporting Qualitative research) Checklist

A checklist of items that should be included in reports of qualitative research. You must report the page number in your manuscript where you consider each of the items listed in this checklist. If you have not included this information, either revise your manuscript accordingly before submitting or note N/A.

Topic	Item No.	Guide Questions/Description	Reported on Page No.
**Domain 1: Research team and reflexivity**			
Personal characteristics			
Interviewer/facilitator	1	Which author/s conducted the interview or focus group?	8
Credentials	2	What were the researcher's credentials? E.g. PhD, MD	8
Occupation	3	What was their occupation at the time of the study?	8
Gender	4	Was the researcher male or female?	8
Experience and training	5	What experience or training did the researcher have?	8
Relationship with participants			
Relationship established	6	Was a relationship established prior to study commencement?	8
Participant knowledge of the interviewer	7	What did the participants know about the researcher? e.g. personal goals, reasons for doing the research	8
Interviewer characteristics	8	What characteristics were reported about the inter viewer/facilitator? e.g. Bias, assumptions, reasons and interests in the research topic	8
**Domain 2: Study design**			
Theoretical framework			
Methodological orientation and Theory	9	What methodological orientation was stated to underpin the study? e.g. grounded theory, discourse analysis, ethnography, phenomenology, content analysis	6-7
Participant selection			
Sampling	10	How were participants selected? e.g. purposive, convenience, consecutive, snowball	7
Method of approach	11	How were participants approached? e.g. face-to-face, telephone, mail, email	7
Sample size	12	How many participants were in the study?	9
Non-participation	13	How many people refused to participate or dropped out? Reasons?	9
Setting			
Setting of data collection	14	Where was the data collected? e.g. home, clinic, workplace	8
Presence of non-participants	15	Was anyone else present besides the participants and researchers?	8
Description of sample	16	What are the important characteristics of the sample? e.g. demographic data, date	9-10, Table 1
Data collection			
Interview guide	17	Were questions, prompts, guides provided by the authors? Was it pilot tested?	8, Supplement
Repeat interviews	18	Were repeat inter views carried out? If yes, how many?	8
Audio/visual recording	19	Did the research use audio or visual recording to collect the data?	8
Field notes	20	Were field notes made during and/or after the inter view or focus group?	8
Duration	21	What was the duration of the inter views or focus group?	8
Data saturation	22	Was data saturation discussed?	10
Transcripts returned	23	Were transcripts returned to participants for comment and/or	8

Topic	Item No.	Guide Questions/Description	Reported on Page No.
		correction?	
**Domain 3: analysis and findings**			
Data analysis			
Number of data coders	24	How many data coders coded the data?	7
Description of the coding tree	25	Did authors provide a description of the coding tree?	9, see referen
Derivation of themes	26	Were themes identified in advance or derived from the data?	9
Software	27	What software, if applicable, was used to manage the data?	9
Participant checking	28	Did participants provide feedback on the findings?	9
Reporting			
Quotations presented	29	Were participant quotations presented to illustrate the themes/findings? Was each quotation identified? e.g. participant number	9-14, Table 2.
Data and findings consistent	30	Was there consistency between the data presented and the findings?	9-14
Clarity of major themes	31	Were major themes clearly presented in the findings?	9-14
Clarity of minor themes	32	Is there a description of diverse cases or discussion of minor themes?	N/A

Developed from: Tong A, Sainsbury P, Craig J. Consolidated criteria for reporting qualitative research (COREQ): a 32-item checklist for interviews and focus groups. *International Journal for Quality in Health Care*. 2007. Volume 19, Number 6: pp. 349 – 357

Once you have completed this checklist, please save a copy and upload it as part of your submission. DO NOT include this checklist as part of the main manuscript document. It must be uploaded as a separate file.

<u>**Supplement 1: Interview Guide for Patients**</u>

I. Introduction

- How many kidney transplants have you had?
- How long ago did you receive your kidney transplant? Was your donor living or deceased?
- How did the transplant impact your life?
- In your experience, what were the biggest differences between life before and after transplant?

II. Experience of Graft Failure

1. Could you tell me about your experience of losing the kidney transplant?
Probes:

- When did it become clear to you that the transplant was failing? Could you tell me about that?
- What did you understand about why you lost your transplant?

2. How did losing the kidney transplant affect your life?
Probes:

- What were you feeling as you were losing the transplant?
- What would you describe as the most difficult part of the experience?
- How did experiencing graft loss affect your life on the day-to-day?
- Did losing the graft change how you feel about your life (or your illness)?
- Did it change how you think about the future?
- Did losing your transplant impact the way you thought about death and mortality? If so, how?
- If applicable, what impact did losing the transplant have on your living donor?

3. Can you describe in more detail what happened next for you, after you lost the transplant?
Probes:

- What were your treatment options after losing the transplant?
- When were your treatment options discussed with you, after the graft started to fail?
- Could you tell me about that?
- When was re-transplantation introduced to you as an option? Could you tell me about
- that?

Questions for patients pursuing re-TX

- Why did you decide to pursue re-transplantation? How clear was it that you were
- interested in another transplant?
- What factors affected your decision?
- Is there anything that concerned you about pursuing another transplant?
- Once you decided to pursue retransplantation, what were the next steps?
- How is the process different from the steps you took to have access to a transplant the
- first time?
- How was/has the process of evaluation for eligibility (been) different this second time?
- In your experience, is there anything that makes it different from the first time?
- Is there anything else that made it difficult for you to pursue getting another transplant?
- (if yes to the question above) How did you deal with these barriers?
- Are you currently re-listed? How long has it been?
    - (if waiting for eligibility) To your knowledge, what would prevent your eligibility for a second transplant? Have you encountered any obstacles?

- (if waiting for eligibility) What kind of information have you been given about your eligibility for retransplantation?
- (If re-listed) What has this waiting period been like for you? How is it different from waiting for your first transplant?
- (If already re-transplanted) Tell me about your second transplant. How was it differentfrom the first? What are the main challenges of having a second transplant?

Questions for patients who are not eligible

- When and how did you learn that you were not eligible for a re-TX? How was it discussed with
- you?
- What were you feeling?

Questions for patients who are choosing not to pursue re-TX

- Why did you decide not to purse re-TX?
- How do you feel about your decision? Was it a difficult decision?
- Did you consider re-TX?

4. How would you describe your current state of health? How are you feeling?

Probes :

- How would you compare your health now with how you felt at other stages of your illness (pretransplant,
- post-transplant).
- What healthcare you are currently receiving? What treatments are you receiving?
- Are there any additional health services that are you currently using?
- If services are mentioned, how are they helping you? If none mentioned, are there services that
- you think could be helpful?

5. What are the things that have helped you throughout your experience of losing your transplant up until now?

- Can you describe your support system? What does it consist of?
- How supported have you felt by healthcare services?
- By the transplant team? (other teams?)
- By family/friends/caregivers?
- Were you offered any resources to help you during this period of graft loss?
- [if applicable] How supported did you feel by health services to be retransplanted?

6. What have you learned through your experiences?

- Are there things you know now that you wish you had known before you lost your graft?
- Is there anything you would have done differently?
- Is there anything you wish others had done differently?
- How has experiencing graft loss changed you?
- How did it change the way you thought about a « succesful » transplant? What does a
- successful transplant mean to you? What outcomes matter to you the most?

7. What were you told about graft loss before you received the kidney transplant?

Probes :

- Do you recall if the subject was ever brought up by your doctor? (were you informed of risk of
- graft failure?)
- Was it addressed post-transplant? How?
- What were your personal expectations about the outcome of your transplant?
- Did you think a lot about the possibility of graft failure? Why or why not?

- Did you feel prepared for graft failure? Did you find that you needed to make psychological
- adjustment as it was happening?

8. When your transplant started failing, what kind of information were you provided with?
Probes :
- Was there information you would have liked to have but did not receive?
- How was the subject of your transplant failing approached by healthcare professionals? Could
- you explain how they discussed it with you?

9. In your experience, what was the transition like between losing the graft and returning to dialysis?
Probes:
- Were there any challenges?
- Is there anything you think could have been done better to make the transition
- smoother?
- What did the care team do to make you feel guided and supported?
- Did you experience any challenges or difficulties with communication with your care
- team(s) during transition?

III. How to Improve Care
10. What could have improved your experience of losing your transplant?
Probes:
- What has been done well?
- Is there anything that you think could have been done better?
- What is an ideal care team, what would that look like for you?
- How satisfied are you with the health services available to you?

11. If you were to advise a close friend or family member going through loss of a kidney transplant, what would you share about your experience that could help them navigate the experience?
- What would you recommend they think about to improve their experience?

12. If you were to advise a health care professional about the needs of patients experiencing loss of a kidney transplant what would you recommend they should think about and do?
- From your perspective, what should HCPs be aware of?

Is there anything else you would like to add? Anything I may have missed?
Thank you very much for your time.

Table S1: Themes and Representative Quotes
**Loss of Control**<br><br>*I mean I did what I was asked. I took my meds when I was supposed to, showed up for my appointments. I don't really think there's anything I could have changed that would have changed the outcome*. (P5)<br><br>*They've biopsied the kidney twice already and done . . . a number of MRIs and scans and just different things to see if there's an issue . . . and have found nothing, there's no reason for it, so they just assume it's the kidney . . . it just isn't working right . . . I mean there's nothing I could do about the kidney that ended up in me*. (P18)<br><br>*And [doctor] said, "I don't think it will last a year. I think we've got it as far as it can go." And I remember thinking, "You've worked very hard to get me this far." They work very, very hard. And you can't get blood out of a stone. We tried as best as we could and now it's done*. (P4)<br><br>*I believe that the doctors and the hospital that performed my transplant, I believe they did everything they were supposed to do . . . That's just life, that's nature, they don't have a choice, they can't perform miracles, they are not God*. (P13)
**Loss of Coherence**<br><br>*Well obviously you expect that it's going to work, right? And you'll be able to go back to a normal life. And when that doesn't happen it's how can I describe it? It's devastating, right? Because you want it to work, right? You want it to work. And you're given the hope that kidney transplants work. You know? They fail very rarely. You know? The advancement that's been done in the . . . antirejection medication . . . the procedures and everything. They make you believe that it's an easy-peasy surgery and that it's going to work. But it' not the case . . . because . . . you are really unaware of what could happen.* (P8)<br><br>*Having to tell people that it didn't work, you know, [family and friends] were so optimistic when they heard that I was getting a transplant, but they weren't ready to hear that it didn't work. And so you got to tell this story over and over again . . . and that was pretty hard . . . there was no one in my community who had gone through something like this before me . . . it was so unfamiliar. I guess to everyone. Unfamiliar and uncomfortable*. (P15)<br><br>*I didn't feel unwell. I felt normal. If I hadn't done the blood tests, I would have never known the graft was failing . . . I would have never known there was a problem with the graft. Because I felt normal . . . I felt so good with the transplant. I was functioning like everyone else. I never thought I would go back to dialysis*. (P13)<br><br>*I was in denial for sure about, like, the way that I felt and how bad I felt. I think, like, the physicians . . . basically they were like, "OK, you're going to have to let us know when you feel ready, when you feel like you need dialysis." And like even though my rational brain says like "yes, it's time" or "you're getting close," my emotional brain was, like, in complete denial that this was my reality*. (P10)

I was trying to fight this tooth and nail, like not to go on dialysis. So I was pushing it off . . . and I was in the clinic . . . and one of the doctors walked by me and he looked at me and said, "I can just see by looking at you that you need to go downstairs right now, get a catheter put in and start dialysis today." . . . and that was kind of a shock because I was stubborn, I just didn't want to, you know. (P21)
**Loss of Certainty**<br><br>*I have always had a hard time imagining my future. Like, not since I was a kid but since diagnosis. It's just so fragile that, like, I have a really hard time imagining myself five, ten years in the future because you really have no idea what your health is going to be like, or like what's going to be going on.* (P10)<br><br>*I found the hardest part was knowing if I was on the list or not for a cadaveric transplant. Like, you have to meet these criteria. And I was never sure . . . because I'd say "Oh, am I on the list?" "No, you're not." And then another person would say, "Oh, yes you are."* (P21)<br><br>*Right now I have no clue if I should drop all the things related to getting a transplant because I can't get one*. (P3)<br><br>*I remember definitely some hesitation [about retransplantation] because I'm like, you know, feel like I was kind of burned the first time. Like I remember my mom and I having some discussions because I'm like I don't know, like dialysis for 20 years doesn't sound great either . . . So I know for myself it was like which is better because once you get like stabilized on dialysis it just becomes a part of life . . . like manageable I guess . . . but at some point you know you have to realize that going to a machine three times a week is just not really a way to live so that would have probably put me over the edge*. (P20)<br><br>*So you go in optimistically. But for a surgery like that, no matter who you are, there's a little bit of apprehension. And you don't know. If it fails you'll be worse off than when you started because you'll have gone through the trauma of surgery and then still be on dialysis*. (P9)
**Loss of Hope**<br><br>*I would like to have a kidney again, I would like to be able to move forward towards something else . . . I would like to get better, but it's like I don't believe in it anymore . . . I've had two types of rejection, so it makes me less eager to experience a third one, but the idea of having a kidney again and living my life well . . . creating a cushion for my retirement and trying to build a life for the future, for sure that's what I really want. Because right now, staying on dialysis, I don't see how I am going to build that. The way things are right now, it's not working*. (P1)<br><br>*I don't have hope that anything is going to happen. Like the next phone call is not going to be like, "Hey, we have a kidney for you" because I am not active [on the list]. But like I don't have that hope, which is the most difficult part right now, I think because I don't see an end to having to go in-unit three times a week to have the little lifeblood sucked out of me*. (P19)

In the evening, when I finish dialysis and go back home, it's like sometimes you wonder if you . . . [don't] wake up tomorrow morning, and you know it's over, you won't have to go back to dialysis again and you'll rest. I am not someone who seeks death, but if it came, I wouldn't tell it to go back home. (P13)

It wasn't like I was depressed . . . I was just really sick all of the time. And some people will say, "Yeah, well, you're depressed." But you're just fed up. You're sick and tired of being sick and tired all of the time. It's like, you know, somebody in jail that, you know, they decide to end it. You know? They say, "There's no hope for me. Why am I here?" . . . And that's how I was feeling . . . there's a pamphlet from one of the clinics that says "choosing to stop dialysis." So I am not the only person to do this. And many have done it. They said, "That's enough. Thank you, everybody, but I am not coming back." (P9)

Loss of Quality of Life

I am someone who lives day by day . . . when you are on dialysis you have no goals. You live, you wait . . . you can't go anywhere and you're life is completely messed up. (P13)

It's always like, why am I here? Why do I have to be here? Like I remember there was a guy in [Dialysis Location], a really nice guy . . . they used to call him Grumbles because he would just be the grumpiest person who ever came in. Like, he hated being there. And it's because you feel terrible, right? Like it's just the worst . . . Ah, man. Yeah I even remember when I was a kid there was a guy named Lloyd and they would like have to constantly call him and get the police to go find him and make him come to dialysis. (P20)

When I was losing the transplant, I was worried about my job, you know, like not being able to do it, or them replacing me.. . . I mean they can't describe how tired you are when your kidneys are not working . . . it's a different level of tired. (P21)

And as far as physical feeling you get to the point where you just want to lie down and not do anything . . . And it was devastating because – see my voice is cracking – because you know I really enjoyed [my] job and I really enjoy food and then OK, job you can't do, you can't eat the food that you like best and you just don't feel like cooking or eating anyway. (P9)

Losing the graft has changed my perception of life due to the fact of not working anymore . . . Because we didn't have much money and I live quite far from the [Hospital], so for all the transportation to [Hospital], we often went without food in order to put gas in the car, to pay for transportation, to go to the hospital three times a week for hemodialysis . . . or appointments for exams. It for sure changed our quality of life . . . It's really difficult financially, and I would say it causes huge financial stress for people. (P7)

I remember the dietician coming in and asking me . . . "how are you eating?" And I said, "Well, not really. Well, like I can't really afford to even buy food right now." (P16)

Loss of Transplant Team

And I feel, "My team's gone. Oh my god, I am all by myself." I felt like that for a little while. (P4)

February I got COVID and I was in the hospital for 37 days. So when I had COVID it was really bad . . . and the creatinine was going up . . . about 400-500 so [doctor at hospital said] "Okay well you have to go on dialysis" . . . and it went from everything going really well to everything going bad . . . and my thought it was "Oh my, what a big disappointment." . . . And as far as losing the kidney it would have been nice if – and I am sure [transplant doctor] knew that I was there [in the hospital] – if he would actually physically come an have a discussion. It never happened . . . It would've been nice to see [transplant team]. (P2)

Normally when you have a failed graft, [the transplant team] . . . doesn't see you anymore and you get transferred to just regular nephrology. .... I know there is a real problem with communication and accountability from the nephrologist side on following up on different things, because there is no assigned nephrologist to a patient. It's just whoever is in clinic that week . . . And, like, I don't know what their handover looks like, but nobody knows anything about me . . . Like I have to talk about my case again. And it's like there's just too many moving parts in my specific case to talk about it with anyone because like -I don't know- like I just feel like it would be hard to keep up with, and there's just like way too many parts to get anything done in an efficient manner. (P10)

My care team, right now, I would consider the Home Dialysis Unit. I have no contact with the transplant department at the moment. (P4)
